# Special Issue “Health and Performance through Sports at All Ages 2.0”

**DOI:** 10.3390/jfmk9010036

**Published:** 2024-02-23

**Authors:** Gianpiero Greco

**Affiliations:** Department of Translational Biomedicine and Neuroscience (DiBraiN), University of Study of Bari, 70124 Bari, Italy; gianpiero.greco@uniba.it

This Special Issue, “Health and Performance through Sports at All Ages 2.0”, aimed to investigate the impact of physical activity and sport on health and human performance across all age groups. Previous research has shown that engaging in sports activities offers numerous health benefits, including improved cardiovascular health, enhanced musculoskeletal strength, and weight management [1]. Physical and sports activity positively impacts the mental and physical health and behaviors of adults [2], the physical fitness of children [3] and adults with behavioral problems [4], the cognitive and social–emotional domains [4,5], and the body image of adolescents [5]. For older adults, engaging in sports activities contributes to healthy aging by preserving cognitive function, mobility, and independence [6]. Additionally, sports participation among children and adolescents promotes healthy growth and development, instils lifelong habits of physical activity, and reduces the risk of obesity and related health issues [7].

During childhood and adolescence, physical activity is crucial for healthy growth and development. Regular exercise helps children build strong muscles and bones, maintain a healthy weight, and develop essential motor skills and coordination [8]. Moreover, participation in sports and physical activities fosters social skills, such as teamwork, leadership, and communication, which are essential for overall development [9].

Physical activity remains essential in adulthood for maintaining health and optimizing performance. Regular exercise reduces the risk of chronic diseases, such as heart disease, diabetes, and certain cancers [1]. It also helps manage weight, improve cardiovascular health, and enhance muscular strength and endurance [10]. Additionally, staying active promotes mental well-being, reduces stress, and improves sleep quality, leading to a better overall quality of life [11].

As individuals age, physical activity becomes even more critical for maintaining health and independence. Regular exercise helps older adults preserve muscle mass and bone density, reduce the risk of falls and fractures, and manage chronic conditions, such as arthritis and osteoporosis [12]. Moreover, staying physically active in old age promotes cognitive function, reduces the risk of dementia, and enhances overall mobility and quality of life [13].

In addition to its impact on health, physical activity plays a vital role in optimizing performance across all age groups. Participation in sports and physical activities enhances athletic performance, improves motor skills and coordination, and fosters a competitive spirit [7]. Moreover, regular exercise helps individuals set and achieve goals, build resilience, and overcome challenges, both on and off the field [14].

Despite the benefits of physical activity, several barriers and challenges can hinder participation at all ages. These may include lack of time, access to facilities, motivation, and physical limitations [15]. Moreover, societal factors such as socioeconomic status, cultural norms, and environmental factors can also influence levels of physical activity across different age groups [16,17].

The articles included in this Special Issue have contributed to expanding our knowledge in this field with new scientific evidence to satisfy the needs of the population of all ages practicing physical activity and sports.

Nineteen manuscripts were submitted for consideration to this Special Issue, and all of them were subjected to the rigorous *JFMK* review process. In total, ten papers were finally accepted for publication and inclusion in this Special Issue (nine articles and one systematic review). The contributions are listed below:

1. Grigoletto, A.; Mauro, M.; Toselli, S. Evaluation of the Effectiveness of a Nordic Walking and a Resistance Indoor Training Program: Anthropometric, Body Composition, and Functional Parameters in the Middle-Aged Population. J. Funct. Morphol. Kinesiol. 2023, 8, 79. https://doi.org/10.3390/jfmk8020079;
2. Bongiovanni, T.; Rossi, A.; Genovesi, F.; Martera, G.; Puleo, G.; Orlandi, C.; Spedicato, M.; Iaia, F.M.; Del Vescovo, R.; Gallo, S.; et al. How Do Football Playing Positions Differ in Body Composition? A First Insight into White Italian Serie A and Serie B Players. J. Funct. Morphol. Kinesiol. 2023, 8, 80. https://doi.org/10.3390/jfmk8020080;
3. Karatrantou, K.; Papavasiliou, T.; Batatolis, C.; Vasilopoulou, T.; Ioakimidis, P.; Gerodimos, V. A Chair-Based Music–Kinetic Combined Exercise Program as an Alternative Approach for Increasing Health, Functional Capacity, and Physical Fitness Indices in Middle-Aged Pre-Menopausal Women. J. Funct. Morphol. Kinesiol. 2023, 8, 81. https://doi.org/10.3390/jfmk8020081;
4. Jagomast, T.; Mohr, T.; Axt, P.N.; Mortensen, K.; Brinkmann, F.; Weckmann, M.; Ring, G.; Reppel, M.; Drömann, D.; Franzen, K.F. Effects of Physical Activity in the High School Curriculum on Cardiovascular Health, Cognitive and Physical Performance. J. Funct. Morphol. Kinesiol. 2023, 8, 101. https://doi.org/10.3390/jfmk8030101;
5. Tsartsapakis, I.; Gerou, M.; Zafeiroudi, A.; Kellis, E. Transversus Abdominis Ultrasound Thickness during Popular Trunk–Pilates Exercises in Young and Middle-Aged Women. J. Funct. Morphol. Kinesiol. 2023, 8, 110. https://doi.org/10.3390/jfmk8030110;
6. Crawford, D.A.; Heinrich, K.M.; Haddock, C.K.; Poston, W.S.C.; Day, R.S.; Kaipust, C.; Skola, B.; Wakeman, A.J.; Kunkel, E.; Bell, A.; et al. A Single, Multimodal Exercise Tolerance Test Can Assess Combat Readiness in Army-ROTC Cadets: A Brief Report. J. Funct. Morphol. Kinesiol. 2023, 8, 152. https://doi.org/10.3390/jfmk8040152;
7. Grigoletto, A.; Mauro, M.; Toselli, S. Differences in Body Composition and Maturity Status in Young Male Volleyball Players of Different Levels. J. Funct. Morphol. Kinesiol. 2023, 8, 162. https://doi.org/10.3390/jfmk8040162;
8. Rodrigues, F.; Jacinto, M.; Antunes, R.; Monteiro, D.; Mendes, D.; Matos, R.; Amaro, N. Comparing the Effects of Multicomponent and Concurrent Exercise Protocols on Muscle Strength in Older Adults. J. Funct. Morphol. Kinesiol. 2024, 9, 3. https://doi.org/10.3390/jfmk9010003;
9. Greco, G.; Centrone, C.; Poli, L.; Silva, A.F.; Russo, L.; Cataldi, S.; Giustino, V.; Fischetti, F. Impact of Coastal Walking Outdoors and Virtual Reality Indoor Walking on Heart Rate, Enjoyment Levels and Mindfulness Experiences in Healthy Adults. J. Funct. Morphol. Kinesiol. 2024, 9, 11. https://doi.org/10.3390/jfmk9010011;
10. Olasagasti-Ibargoien, J.; Castañeda-Babarro, A.; León-Guereño, P.; Uria-Olaizola, N. Barriers to Physical Activity for Women with Physical Disabilities: A Systematic Review. J. Funct. Morphol. Kinesiol. 2023, 8, 82. https://doi.org/10.3390/jfmk8020082.

In Contribution 1, the authors wanted to investigate new strategies to combat sedentary behavior in the middle-aged population by making use of green spaces. They compared the effectiveness of a period of outdoor training (Nordic walking) with indoor resistance training in a non-clinical middle-aged population based on anthropometric characteristics, body composition, and functional parameters. The Nordic walking group showed a higher elevation in muscle mass and a higher decrease in fat parameters than the indoor resistance training group. However, these two types of training could represent a good strategy to remain active and prevent sedentary behaviors.

The authors of Contribution 2 investigated how playing positions differ in specific body composition variables in professional soccer players concerning specific field zones and tactical lines. These authors analyzed the following positions: goalkeepers (GKs), central backs (CBs), fullbacks (FBs), central midfielders (MIDs), wide midfielders (WMs), attacking midfielders (AMs), second strikers (SSs), external strikers (ESs), and central forwards (CFs), as well as their field zones (central and external) and tactical lines (defensive, middle, and offensive). The GKs and CFs were the tallest and heaviest players, with no differences from each other. Likewise, the GKs and CFs, along with the CBs, were apparently more muscular (for both the upper and lower limbs) and fatter at the same time compared with the other roles. Overall, players of the defensive line (e.g., the CBs and FBs), along with those playing in central field zones (e.g., the CBs, MIDs, AMs, SSs, and CFs), were significantly superior in almost all anthropometric and body composition variables to those of middle and offensive line and external zones, respectively.

In Contribution 3, the authors considered the chairs that have been widely used as cheap, easily accessible, safe, and effective training means in different settings (e.g., in gyms, houses, workplaces, and in rehabilitation). These authors investigated the effectiveness of a 10-week chair-based music–kinetic-integrated combined exercise program (aerobic dance, flexibility, coordination, and strength exercises with body weight or auxiliary means) on the health, functional capacity, and physical fitness indicators of middle-aged pre-menopausal women. The exercise group showed significantly reductions in body fat, blood pressure, the time during the timed up-and-go test, heart rate, and the rate of perceived exertion, while increasing respiratory function, flexibility, balance, maximal handgrip strength, and endurance. These authors concluded that the chair-based combined music–kinetic exercise program was effective and could be safely used in different settings to improve health, functional capacity, and physical fitness in middle-aged women.

Although it is well-known that cardiovascular health at a young age has implications for preventing cardiovascular disease and is associated with improved physical and cognitive performance during the aging process [1], the authors of Contribution 4 wanted to address the problem of physical activity interventions at school that are overlooked. They compared groups of high school students, stratified by the level of physical activity in their high school curriculum and downtime. Regarding cognitive skills, extracurricular physical activity improved the number of connection tests in male participants. For physical performance, female students with a sports-focused curriculum were faster in the 3 km run. Concerning arterial stiffness, the measurements yielded a lower mean arterial pressure and aortic pulse wave velocity in male students with a sports-focused curriculum. These authors suggested that extracurricular physical activity and enrolment in a sports-focused curriculum may be associated with lower cardiovascular risk due to lower levels of arterial stiffness and better physical and cognitive abilities.

It Is also known that the transversus abdominis is a core muscle that contributes to functional mobility and lumbar stability. In Contribution 5, the authors aimed to compare the changes in transversus abdominis thickness during different Pilates exercises (basic position, hundred, hip roll, side plank, and dead bug) and identify the exercise that elicited the greatest transversus abdominis activation. Their findings suggested that the dead bug exercise is the most effective for enhancing transversus abdominis activation among the Pilates exercises that were tested. The basic position and the hundred exercises can be used as warm-up exercises before performing more challenging exercises, such as the hip roll, the side plank, and the dead bug. The sequence of exercises can be similar for both young and middle-aged women.

In Contribution 6, the authors, through their research on tactical populations, sought to design and evaluate a single, multimodal exercise tolerance test capable of serving as a time-efficient proxy measure of combat readiness. It is well known that the Army Combat Fitness Test is a multi-event assessment battery designed to determine the combat readiness of U.S. Army personnel. Unfortunately, for Reserve Officers’ Training Corps programs, the logistical demands of collegiate life make repeated administration of the Army Combat Fitness Test challenging. The findings suggested that the multimodal exercise tolerance test has the potential to provide a means to monitor progress, identify areas for improvement, and guide informed decision making regarding the individualization of cadet combat training plans.

Another interesting piece of research in this Special Issue was carried out on one of the most played sports, namely volleyball, which is an intermittent team sport that requires specific anthropometrical and physical characteristics for winning performance. The authors of Contribution 7 evaluated the maturity status of the young male players of eight volleyball teams and observed differences in anthropometric characteristics and body composition. Their findings showed that young volleyball players classified as “early” seemed to show anthropometric characteristics linked to better performance at the tournament (higher height, upper arm and calf muscle area, fat mass percentage, and total fat-free mass). The authors suggested that the results of the study could have practical implications for talent selection, but further studies are needed to better evaluate the effect of maturity status on the characteristics of volleyball players.

A study on the elderly was carried out by the authors of Contribution 8, which compared the effects of a multicomponent exercise program and a concurrent exercise program on muscle strength in community-dwelling elderly subjects. These authors found that while both the multicomponent and concurrent exercise programs were effective in improving muscle strength in community-dwelling older adults, the multicomponent exercise group exhibited superior outcomes compared to the concurrent exercise group across the physical fitness measures. These findings suggest that a multicomponent exercise program may be more beneficial for enhancing muscle strength in this population.

As has been shown by Contribution 1, outdoor exercise in a green space is beneficial for peoples’ well-being. However, limited studies have compared outdoor and virtual reality indoor physical activities, especially in coastal settings. Therefore, the authors of Contribution 9 assessed the impact of outdoor coastal walking and indoor walking in a virtual reality simulation with a similar environment on physiological and psychological variables in healthy adults. Their findings suggested that physical activity in immersive technology may lead to physiological loads comparable to those in the outdoor environment. Outdoor walking is more enjoyable than indoor walking and virtual reality indoor walking, but it exhibits a mindfulness response comparable to virtual reality indoor walking. Therefore, virtual reality indoor walking could be an alternative to outdoor walking for those who cannot engage in outdoor activities for various reasons.

Finally, the systematic review of Contribution 10 aimed to identify the barriers faced by women with physical disabilities in practicing sports. The barriers to physical activity for women with physical disabilities are multiple and complex, and span multiple dimensions. This review identified different barriers, including (i) personal barriers such as age, fatigue, loneliness, lifestyle, or simply being a woman may limit participation in any physical activity; (ii) motivation and other psychological factors such as fear, the perception of not being able to engage in physical activity, or a negative self-perception; (iii) any management barriers, such as poor accessibility or lack of adaptations to sports centers, lack of transport to sports facilities, lack of communication between professionals, and poor organizational management; (iv) very often, training staff are not adequately trained to adapt physical activity or programs to users’ needs; (v) the financial costs of sporting activities as this barrier can have a crucial impact on participation in sport; (vi) men received more support from family and friends than women; and (vii) social attitudes towards people with disabilities: people with disabilities who perceive stereotyping to a high degree also perceive a lower quality of life. Disabled people’s participation in physical activity is directly related to some specific barriers that seem to differ according to their gender. Therefore, the success of participation in physical activities depends not only on the user’s concern but also on an inclusive social environment.

In conclusion, sports participation provides numerous health benefits and contributes to improved performance throughout the lifespan [18,19,20]. Incorporating regular physical activity through sports engagement is essential for promoting overall health, optimizing performance, and addressing age-related challenges. Future research should continue to explore the specific mechanisms underlying the relationship between sports participation and health outcomes, with a focus on developing tailored and adapted interventions to encourage lifelong physical activity engagement. 

Given the great success of the second edition of this Special Issue, we have launched a third edition, for which we hope to receive contributions focusing on the effects of sports and physical activity on health and human performance across all age groups.
